# Supplementary material for: Oncometabolic role of mitochondrial sirtuins in glioma patients
Source: PLoS One. 2023 Feb 21;18(2):e0281840. doi: 10.1371/journal.pone.0281840 (PMC9943017; doi:10.1371/journal.pone.0281840)
Supplement: Attachment — Submitted filename: PONE-D-22-26011.docx [file pone.0281840.s001.docx]

Comments to Authors:

This is an interesting work PONE-D-22-26011 Oncometabolic Role of Mitochondrial Sirtuins in Glioma Patients. which aims to clarify the regulation of mitochondrial sirtuins and related genes in glioma cells. This work lays the foundation for future research in the study of gliomas. To improve your manuscript, some suggestions are listed below:

1) The abstract is quite long and you may risk losing the reader’s attention. I suggest you rewrite it in a more synthetic way in order to just highlight the contents.
2) The introduction turns out to be not very fluid: we appreciate the meticulous characterization of all the studied genes, but we invite you to improve the fluidity of this part.
3) As mentioned in the introduction, mitochondria are fundamental organelles involved in many biological processes like ATP production and apoptosis.
    In glioma cells, the necroptosis process is also present and actively studied. We strongly suggest you read and cite it in your work (DOI: [10.1038/s41420-022-00974-x](https://doi.org/10.1038/s41420-022-00974-x) )
4) We encourage you to double check your manuscript font as some variances were found. We also ask you to specify why OGG12 is sometimes written as OGG1-2α.
5) The resolution of table 2 is too low. It results blurred and pixelated. Please, check and improve the quality of this image.

6) All your figures require major improvements and they are all presented in different sizes, making them visually chaotic.
   6.1) in Figure 1, your graphs are not uniform. Some histograms have bold outlines while other have not. Also make sure your titles on the y axis have all the same character size.
   6.2) In Figure 2, figure 3F, figure 5 and figure 6 are too small and difficult to read. We ask you to make them bigger and clearer for the reader.
   6.3) Similarly, figures 7 and 8 have small tables under the graphs. Please, improve their resolution and size.
7) We recommend you to to standardize the letters indicating the various images of the panels. Some are bigger, while other are smaller and they need to be aligned in order to make your panel cleaner. Furthermore, we advise you to choose the same square shape for all the letters.
