# Supplementary material for: Oncometabolic role of mitochondrial sirtuins in glioma patients
Source: PLoS One. 2023 Feb 21;18(2):e0281840. doi: 10.1371/journal.pone.0281840 (PMC9943017; doi:10.1371/journal.pone.0281840)
Supplement: Attachment — Submitted filename: revision response.docx [file pone.0281840.s002.docx]

Journal Requirements:

When submitting your revision, we need you to address these additional requirements.

1. Please ensure that your manuscript meets PLOS ONE's style requirements, including those for file naming. The PLOS ONE style templates can be found at
   https://journals.plos.org/plosone/s/file?id=wjVg/PLOSOne_formatting_sample_main_body.pdf and <https://journals.plos.org/plosone/s/file?id=ba62/PLOSOne_formatting_sample_title_authors_affiliations.pdf>

We go through the manuscript and arrange according to Plos One style requirement as per suggestion of the worthy editor.

2. We noticed you have some minor occurrence of overlapping text with the following previous publication(s), which needs to be addressed:

https://www.futuremedicine.com/doi/10.2217/fon-2021-0264
https://www.mdpi.com/2072-6694/9/7/90/htm
https://www.futuremedicine.com/doi/10.2217/fon-2019-0365
https://www.frontiersin.org/articles/10.3389/fonc.2018.00622/full

In your revision ensure you cite all your sources (including your own works), and quote or rephrase any duplicated text outside the methods section. Further consideration is dependent on these concerns being addressed.

Overlapping texts have been rephrased as per suggestion of the worthy editor.

3. Please provide additional details regarding participant consent. In the ethics statement in the Methods and online submission information, please ensure that you have specified (1) whether consent was informed and (2) what type you obtained (for instance, written or verbal, and if verbal, how it was documented and witnessed). If your study included minors, state whether you obtained consent from parents or guardians. If the need for consent was waived by the ethics committee, please include this information.

If you are reporting a retrospective study of medical records or archived samples, please ensure that you have discussed whether all data were fully anonymized before you accessed them and/or whether the IRB or ethics committee waived the requirement for informed consent. If patients provided informed written consent to have data from their medical records used in research, please include this information.

Additional information regarding the participant consent and required detail of ethical review board have been added and highlighted red in Materials and methods section as per suggestion of the worthy editor.

4. Thank you for stating the following in the Acknowledgments Section of your manuscript: 
"This study was supported by the grants from higher education commission of Pakistan as well as the COMSATS institute of information Technology Islamabad."

We note that you have provided funding information that is not currently declared in your Funding Statement. However, funding information should not appear in the Acknowledgments section or other areas of your manuscript. We will only publish funding information present in the Funding Statement section of the online submission form.

Please remove any funding-related text from the manuscript and let us know how you would like to update your Funding Statement. Currently, your Funding Statement reads as follows:

"The author(s) received no specific funding for this work."

Please include your amended statements within your cover letter; we will change the online submission form on your behalf.

Funding information has been corrected and removed from the manuscript as per suggestion of the worthy editor.

Correct funding statement has been added in cover letter as per suggestion of the worthy editor.

5. PLOS requires an ORCID iD for the corresponding author in Editorial Manager on papers submitted after December 6th, 2016. Please ensure that you have an ORCID iD and that it is validated in Editorial Manager. To do this, go to ‘Update my Information’ (in the upper left-hand corner of the main menu), and click on the Fetch/Validate link next to the ORCID field. This will take you to the ORCID site and allow you to create a new iD or authenticate a pre-existing iD in Editorial Manager. Please see the following video for instructions on linking an ORCID iD to your Editorial Manager account: <https://www.youtube.com/watch?v=_xcclfuvtxQ>

ORCID information has been added as per suggestion of the worthy editor.

---------------------------------------------------------------------------------------------------------

Reviewer #1:

1) The abstract is quite long and you may risk losing the reader’s attention. I suggest you rewrite it in a more synthetic way in order to just highlight the contents.

Abstract has been rewritten in more comprehended form (highlighted red) as per suggestion of the worthy review.

2) The introduction turns out to be not very fluid: we appreciate the meticulous characterization of all the studied genes, but we invite you to improve the fluidity of this part.

Introduction section has been thoroughly checked/corrected (highlighted red) and fluidity of the introduction section has been improved as per suggestion of the worthy reviewer.

3) As mentioned in the introduction, mitochondria are fundamental organelles involved in many biological processes like ATP production and apoptosis.
In glioma cells, the necroptosis process is also present and actively studied. We strongly suggest you read and cite it in your work (DOI: 10.1038/s41420-022-00974-x )

Suggested information has been added in introduction section (highlighted red) as per suggestion of the worthy reviewer.

4) We encourage you to double check your manuscript font as some variances were found. We also ask you to specify why OGG12 is sometimes written as OGG1-2α.

Manuscript has been thoroughly checked and correct term (OGG1-2α; mitochondrial subunit of OGG1)) has been added throughout the manuscript as per suggestion of the worthy reviewer.
5) The resolution of table 2 is too low. It results blurred and pixelated. Please, check and improve the quality of this image.

Quality and pixel of Table 2 has been improved for more clarification as per suggestion of the worthy reviewer.

6) All your figures require major improvements and they are all presented in different sizes, making them visually chaotic.

All suggested changes have been made in figures as per suggestion of the worthy reviewer.
6.1) in Figure 1, your graphs are not uniform. Some histograms have bold outlines while other have not. Also make sure your titles on the y axis have all the same character size.

Figure 1 has been improved and all histograms are made uniform with respect to outlines. Same size character has been used in titles of Y axis as per suggestion of the worthy reviewer.

6.2) In Figure 2, figure 3F, figure 5 and figure 6 are too small and difficult to read. We ask you to make them bigger and clearer for the reader.

Figure 2, Figure 3F, Figure 5 and Figure 6 have been improved with respect to size and clarity as per suggestion of the worthy reviewer.

6.3) Similarly, figures 7 and 8 have small tables under the graphs. Please, improve their resolution and size.

Tables associated with Figures 7 and 8 have been improved with respect to size and resolution as per suggestion of the worthy reviewer.

7) We recommend you to to standardize the letters indicating the various images of the panels. Some are bigger, while other are smaller and they need to be aligned in order to make your panel cleaner. Furthermore, we advise you to choose the same square shape for all the letters.

All images have been improved and presented in more uniform way as per suggestion of the worthy reviewer. Figures have been removed from the main manuscript file and uploaded separately as till files as per instruction of the worthy editor.

---------------------------------------------------------------------------------------------------------------

Reviewer #2:

1. The authors have submitted a ms aimed at demonstrating deregulation of several genes resulting in increased oxidative stress and DNA damage related to worse survival of patients with glioma. The authors also provide evidence that mitochondrial sirtuins and sirtuin-related genes can play a role of diagnostic and prognostic biomarker for this neoplasm.

Previous studies reporting the diagnostic/prognostic values of mitochondrial sirtuins in brain tumor and other cancers, has been added in discussion section (highlighted red) as per suggestion of the worthy reviewer.

2. However the authors should provide more details on some aspects of their work:
   Can epileptic patients be considered "healthy controls" with no abnormality affecting the deregulation of genes and the pathways the authors show to be involved ? In which occasion and why the samples have been obtained from the epileptic patients ?

Since brain tumor surgery is restricted to tumor section only and the surgeon attempts to remove the tumor without damaging vital brain tissue. The surgeon may remove as much of the tumor as possible without taking any distant region. Due to these restrictions, we could not take the adjacent normal tissue sections to be used as controls like other cancers. Controls were taken from the brain surgeries of epilepsy patients or from other surgeries of brain.

Previous studies have reported that epileptic patients’ sections can be taken/used as controls (doi: 10.1016/j.cell.2020.05.007: doi: 10.4049/jimmunol.1301966.).

Our results have also shown the significant difference between the expression pattern of selected genes/pathway in glioma patients and epileptic patients section taken as controls.

3. Why the authors did not carry on any seahorse analysis to achieve a better understanding of the metabolic abnormalities ?

Oxygen consumption rate is measured by most advanced technology of seahorse based bioenergentic analysis. We were unable to perform this analysis due to unavailability of seahorse analyzer and added (highlighted red) this technique in our study limitations in discussion section. However, measurement of ATP and NAD has been performed which is considered a basic step for the measurement of metabolic rate in cell.
